# Meanings of carers’ lived experience of “regulating oneself” in forensic psychiatry

**DOI:** 10.1080/17482631.2022.2094088

**Published:** 2022-06-27

**Authors:** Lars Hammarström, Siri Andreassen Devik, Marie Häggström, Ove Hellzen

**Affiliations:** a Department of Nursing, Mid Sweden University, Sundsvall, Sweden; b Faculty of Nursing and Health Sciences, Nord University, Namsos, Norway

**Keywords:** Forensic mental health, lived experience, nurse-patient relationship, nursing, phenomenological-hermeneutics

## Abstract

**Purpose:**

This study aimed to illuminate the essential meanings of carers’ lived experience of regulating themselves when caring for patients with mental illnesses in forensic inpatient care.

**Methods:**

Qualitative analysis was used to analyse data from narrative interviews with open-ended questions conducted with nine carers, which were analysed using a phenomenological-hermeneutic approach.

**Results:**

Findings revealed three themes, “preserving oneself as a carer,” “building an alliance with the patient” and “maintaining stability in the community.” Carers not only regulated emotions related to patients but also the ward to facilitate a caring climate. For carers, encounters with patients meant facing expressions of suffering that evoked unwanted emotions. Regulating one’s emotions also meant being emotionally touched and facing one’s vulnerability.

**Conclusion:**

Regulating oneself was a strategy used by carers to get closer to the patient and establishing a trusting relationship. Regulating oneself meant becoming aware of one’s shortcomings, not projecting them onto others, which may impair establishing relationships with patients and fulfilling the aim and caring task of forensic psychiatry. This study stresses the importance of carers being guided to manage their conflicting emotions and vulnerabilities and finding courage and an approach that allows a permissive climate of self-reflection.

## Introduction

Forensic psychiatry is synonymous with what is considered to be an environment with a focus on safety and security (Seppänen et al., 2018), where carers are constantly faced with the duality of their profession as being either fostering or caring (Hörberg, 2008). This means they deal with heightened emotions and risk becoming emotionally blunted, which affects motivation and the well-being of both themselves and the patients when caring in a high-security hospital (Hui et al., 2017). Hence, a strategy to manage emotions or “regulate oneself” and not to act upon conflicting emotions is necessary—a clinical strategy and concept that has emerged from a prior study (Hammarström et al., 2019).

Encounters between carers and patients in a forensic setting are mostly spontaneous during daily activities and involve openings for dialogue and opportunities to provide support (Rytterström et al., 2020). However, these encounters could also mean dealing with physical and verbal outbursts from patients, being forced into conditions in which setting limits is considered a part of everyday work (Eivergård et al., 2020) and dealing with uncomfortable situations, sometimes alone (Rytterström et al., 2020). At the same time, establishing a fruitful relationship (Rydenlund et al., 2019) is perceived as emotional labour, meaning that it is necessary to deal with one’s emotions to care for another person (Edward et al., 2017).

The perceptions of aggressive behaviour and reasons for outbursts differ between carers and patients (Berring et al., 2015). Processing a spoken emotion is crucial to apprehend the emotions of others and is important for developing empathy, which may potentially curb violence and aggressive behaviour (Leshem et al., 2020). Emotions that stem from being confronted by patients’ expressions of suffering force carers to unravel and understand these expressions to give an adequate response based on compassion rather than abandoning the patient in a time of need (Hammarström et al., 2020). To deal and cope with these stressful situations, carers use the strategy of controlling their own emotions, regulating themselves to avoid acting on the initial feeling of frustration, fear or anger, remaining in the situation and getting a grip on themselves to feel emotionally ready to handle the situation and patient (Hammarström et al., 2019). Regulation of emotions and arousal mediates behavioural responses to environmental stressors and helps the individual avoid aggressive or disciplinary actions; instead, they can solve the situation using a calm approach (McDonnell et al., 2015).

Dealing with own emotions is a crucial part when working with others (e.g., in psychiatric care) and reflects a process of emotional labour (Hochsild, 1983). Gross (1998) argued an early strategy in this process, which is referred to as emotional regulation, implies suppressing or reappraising one’s emotions. This is strongly correlated with carers’ behaviour towards forensic patients, as they become less antagonistic by reducing self-criticism and increasing self-compassion (Oostvogels et al., 2018). Carers who avoid dealing with and managing their emotions risk becoming distanced, detached or disengaged (Oates et al., 2020).

The emotional burden of caring in a forensic setting means that carers are becoming increasingly vulnerable to burnout (Edward et al., 2017) and must deal with stressful situations characterized by threats, violence and harassment from patients over long periods. This also means that carers must deal with emotions such as frustration, fear, humiliation and disappointment (Hammarström et al., 2019). For this reason, carers must learn and develop further skills that promote recovery and decrease restrictive practices. Controlling one’s emotions could be understood as a strategy for managing and minimizing problematic behaviour (Hejlskov Elvén & Edfelt, 2017) and being able to de-escalate a stressful situation (Barr et al., 2019). Handling own emotions is an essential component in forensic psychiatry, which can lead to negative psychosocial outcomes if not addressed, such as job stress and fatigue (De Castro et al., 2004). The way that carers react to patients’ expressions is largely unknown, especially how they cope with their own reactions in the face of problematic behaviour in clients (Looff et al., 2018). This study, therefore, aimed to illuminate the essential meanings of carers’ lived experience of regulating themselves when caring for patients with mental illnesses in forensic inpatient care.

## Methods

### Design

To gain access to carers’ lived experience and essential meanings of regulating themselves when caring for patients with mental illnesses in forensic inpatient care, a qualitative design was chosen. We developed our understanding through the process and method of phenomenological-hermeneutics according to Lindseth and Norberg (2004). This is a method derived from the idea that we cannot understand what others experience, but we can understand the meaning of their experience (Ricoeur, 1976). This study conforms to the Standards for Reporting Qualitative Research (SRQR) checklist (O´Brien et al., 2014).

### Procedure and setting

Approval for carrying out the interviews was obtained by the head of the clinic. Participants were informed and recruited through email and phone calls; all interviews were carried out with consent. The clinic accommodated approximately 180 employees and 100 patients across eight wards. The clinic is one of Sweden’s larger regional clinics with national admission and is classified as a high-security clinic with one department classified as very high. In Sweden, forensic psychiatric care is provided at 28 forensic psychiatric clinics (Innocenti et al., 2014). Care is governed by the law on forensic psychiatric care (LRV, 1991:1129) and treats the special conditions for people transferred to care by the court due to a criminal sanction. The forensic psychiatric clinics are divided into three security levels (very high, high or acceptable), and if the care of a ward is provided across several premises, each ward must be classified on its own (SOSFS, 2006: 9). The patients were mostly men aged 25–45 years, diagnosed with schizophrenia and transmitted to involuntary inpatient forensic psychiatric care due to some sort of violent crime under the Forensic Mental Care Act (SFS, 1991:1129) rather than being incarcerated within the penal system.

### Participants and data collection

Narrative interviews with open-ended questions were performed with nine participants at a forensic hospital in Sweden during December 2020 to February 2021. The interviews were conducted by the first author, a specialist nurse in psychiatric care and researcher, to achieve high information power as described by Malterud et al. (2015). All of the participants were chosen with a purposive sample of having prior work experience of forensic inpatient care with patients with mental illness. The age of the participating carers ranged from 30 to 66 years (Md = 41.6 years) and their experience of forensic inpatient care ranged from two to 32 years (Md = 12 years). The participants consisted of five males and four females; among these, five were specialist nurses in psychiatric care, one was a registered nurse and three were assistant nurses. In this study, all participants are referred to as “carers” to conceal their identities. The interviews were all conducted digitally with video and audio recording following the restrictions due to the COVID-19 pandemic that prohibited the first author from personally visiting the participants. The interviews lasted between 39 and 51 minutes (Md = 44 min). Participants were encouraged to share their narratives about their lived experiences of regulating themselves when caring for patients with mental illnesses in forensic inpatient care. Later, we asked the following open-ended questions: “Can you tell me about an encounter that was difficult to handle?” “Can you tell me about a difficult encounter with a positive outcome?” “Can you tell me about a difficult encounter with a negative outcome?” These were followed up with the following questions: “How did that make you feel?” “Please tell me more.” “Can you give me an example?” The first author transcribed each interview verbatim.

### Data analysis

In order to illuminate the essential meanings of carers’ lived experience of the phenomenon of regulating oneself, the interviews were transcribed verbatim and analysed using a phenomenological-hermeneutic approach according to Lindseth and Norberg (2004). Which is based on the ideas and thoughts of French philosopher Ricoeur (1976); that is, striving to understand what the text is saying. The method goes through three phases: a naïve understanding, structural analysis and a comprehensive understanding. During the first phase—the naïve understanding—the interviews were read numerous times to get a sense of the text as a whole, resulting in an introductory understanding. The structural analysis is a more precise part of the analysis, with an emphasis on rigour. The analysis involved first dividing the text into meaning units, which later were condensed and reflected upon to form subthemes and themes based upon resemblances and diversities (Table 1). The text is seen as multidimensional, and multiple structural analyses may be performed to disclose various meanings (Lindseth & Norberg, 2004). The last and final step—comprehensive understanding—involves arriving to an understanding of the text as a whole and elucidating the utterance meaning of the participants’ lifeworlds (Ricoeur, 1976). This is based on an interpretation of the naïve understanding, the findings from the structural analysis and the authors’ prior understanding of and reflections on the aim, context and literature of the study (Lindseth & Norberg, 2004).

**Table 1.** Example of structural analysis.

Meaning unit	Condensation	Sub-theme	Theme
*“To let that adrenaline rush come and just accept that feeling of fear and just try to continue to be who you are, not showing how afraid I was. If other patients see me afraid, there is a risk that they become worried. I guess that was what I did, but it was difficult. Because he was so sick and scary”*	Accepting fear and being yourself but not showing fear to not worry other patients is difficult when the patient is scary.	Fighting an Inner Battle	Preserving Oneself as a Carer
*“Sometimes you can get frustrated, but then I usually still try to think about what his nagging stands for. His frustration must probably still be greater. To stop and think about why I get provoked. I have to put the lid on and not show how pissed off or disappointed I get. Then the chance increases that I can handle the situation.”*	Sometimes frustration arise and I remind myself what it stands for, his frustration is probably greater. To reflect on why I get provoked I must put the lid on and not show my emotions. Then I might handle the situation.	Fighting an Inner Battle	Preserving Oneself as a Carer
*“I can feel proud and happy that I did not get upset or short-changed or unwound in a situation. It’s probably about finding a confirmation within myself just as much.”*	Feeling proud and happy that I did not get upset. It is probably about finding confirmation within myself.	Being a Professional	Preserving Oneself as a Carer

### Ethical considerations

Written information and a presentation about the research were presented to each participant. All interviews were conducted after obtaining written and informed consent that was kept and stored by the first author. The study was carried out with the approval of the Swedish Ethical Review Authority (No. 2018/157-31). Involvement in the study was voluntary, and participants could choose to end the interview at any time. The interviews were conducted according to the Declaration of Helsinki (World Medical Association, 2008) and in line with guidelines provided by The Swedish Research Council (2016). Everyone was given contact information for all the authors in case questions should arise. A plan was also established with the head of each ward in case any apprehensive feelings should arise.

## Results

### Naïve understanding

The strategy of regulating oneself was triggered by the experience of being confronted by the patient. Patient expressions varied, but could evoke intuitive emotions such as anxiety, irritation, disappointment, frustration and despair—emotions that were perceived as an obstacle to building trust and establishing contact with the patient. By taking control of one’s own emotions, the intention was to calm down the patient and the situation in order to avoid escalation or triggering a chain of negative reactions that could be harmful to the patient, oneself as well as other carers or people in the environment. Regulating oneself did not only mean meeting the patient’s expression, it also meant interpreting and reacting to the “community” (eg the ward) which meant both influencing and being influenced by the others. This process of regulation presupposed ability to recognize, isolate and predict the consequences of the feelings that arose before they were released.

### Structural analysis

The structural analysis resulted in three themes. The first theme is “Preserving oneself as a carer” with the subthemes “Fighting an inner battle,” “Being a professional” and “Fleeing the situation”—a theme that describes carers’ regulating emotions in relation to themselves. The theme “Building an alliance with the patient” with the subthemes “Experiencing affinity in the unique situation and present” and “Being trustworthy” describes how carers regulate emotions in relation to the patients. The third theme, “Maintaining stability in the community” with the subthemes “Being perceptive to the dynamics in the community” and “Being a safe companion” describes how carers regulate emotions in relation to the entire ward, including patients and other carers (Table 2).

**Table 2.** Overview of themes and subthemes.

Themes	Preserving oneself as a carer	Building an alliance with the patient	Maintaining stability in the community
Subthemes	Fighting an inner battleBeing a professional	Experiencing affinity in the unique situation Being trustworthy	Being perceptive to the dynamics in the communityBeing a safe companion
	Fleeing the situation		

### Preserving oneself as a carer

The theme of preserving oneself as a carer describes carers’ emotional responses by understanding and managing their emotions as their professional role was threatened. It involves being able to regulate one’s emotions by being active and confronting opposite and contradictory emotions, thereby taking control of the situation. This was achieved by overcoming emotions that could sometimes be seen as an obstacle for carers when dealing with stressful situations, which is further described in the three subthemes: “Fighting an inner battle,” “Being a professional” and “Fleeing the situation.”

### Fighting an inner battle

In this subtheme, carers described how they were fighting an inner battle during encounters when being temporarily alone with the patient; situations characterized by threats, violence and nagging behaviour from the patient. Having knowledge and a relationship with the patient made it easier to predict the patient’s actions, thereby addressing the carer’s fear. The closer the carer-patient relationship was and the more information the carers had about previous violent actions, the easier carers were able to foresee the way a situation would develop. This means they could deal with their fear by accepting it but not showing it. Carers emphasized the importance of not showing fear in front of the patient, thereby gaining control of the situation and preventing fear from spreading.

To let that adrenaline rush come and just accept that feeling of fear, and just try to continue to be who you are, not showing how afraid I was. If other patients see me afraid, there is a risk that they become worried. I guess that was what I did, but it was difficult because he was so sick and scary.

Carers also described being affected by nagging behaviour and harassment. In such situations, carers tried to overcome feelings of frustration by trying to interpret the patient’s actions and emotions. Being present in such stressful situations and refraining from acting out on initial emotions, such as disappointment, irritation or anger, meant not revealing their emotions in the present situation. As one carer explained:

Sometimes you can get frustrated, but then I usually still try to think about what his nagging stands for. His frustration must probably still be greater. To stop and think about why I get provoked. I have to put the lid on and not show how pissed off or disappointed I get. Then the chance increases that I can handle the situation.

### Being a professional

Being a professional meant dealing with one’s expectations of being a professional carer, managing expectations of oneself and expectations from other carers and having reasonable expectations of the patient. Expectations of oneself mean being able to control one’s feelings and find pride and confirmation in dealing with a situation and avoiding worries in the department. In their narratives, carers emphasized the importance of doing a good job when emotions took supremacy, staying neutral and refraining from acting on such emotions.

I can feel proud and happy that I did not get upset or short-changed or unwound in a situation. It’s probably about finding a confirmation within myself just as much.

Being professional and experienced means being the one who has worked longer, knows the patient better or is familiar with similar situations. It also means dealing with expectations from others and being the one to take the initiative in a difficult situation. Carers described how being a support for others and being the one knowing how to deal with and act in the situation meant that they felt that they were expected to remain calm and controlled. Being professional also meant having reasonable expectations of the patient’s ability to solve the situation and having a realistic view of their progression instead of overestimating the patient’s ability.

But then I also felt this with the experience, that I was really full of my own fears. I was expected to come in with my peace and at that moment be a support to her … I think reflecting on my own actions is fruitful. It’s about lowering your expectations when you go in to talk to them. That you can see his small and positive advances.

### Fleeing the situation

At times, carers expressed being unable to reappraise overwhelming emotions and instead abandoned and fled the situation. Doing so, they suppressed conflicting emotions and left the patient stranded and alone. In other words, they distanced themselves not only from the situation but also from fear or frustration. For these carers, not being able to cope meant ascribing blame and criticism to themselves, which reduced their sense of well-being.

*“Sometimes I feel I just can’t handle it; I just need to flee from the fear or frustration. Of course, it feels wrong, I know that it only makes it worse for the patient and my colleagues … but it just becomes too much.”*

### Building an alliance with the patient

The theme of building an alliance with the patient describes carers’ lived experiences of emotional responses to the patients’ actions and verbal expressions in the situation, characterized by being available, involved and reliable. Carers emphasized the fact that regulating emotions meant getting closer to the patient and thereby strengthening the carer-patient relationship. Hence, regulating oneself meant striving to find a sustainable relationship with the patient. This is further described in the subthemes “Experiencing affinity in the unique situation” and “Being trustworthy.”

### Experiencing affinity in the unique situation

Handling emotions during a stressful and unique encounter means turning the gaze inwards to adapt to the patient, staying open and accessible and understanding the patients’ needs by realizing one’s weaknesses. The carers experienced affinity in the unique situation and an awareness of being able to open up and share something that is considered personal. Allowing the patient to see past the professional role while remaining available may help carers continue to be in the moment rather than abandoning the patient, and it can help sort out the situation.

I have to take into account that we all have easier or harder patients. Often, we need to be consistent and follow the rules. But sometimes it’s important to be flexible and empathetic to not let them down. In a confrontation, it is important that I open up and make myself available for the patient.

Sharing something about oneself and opening up meant getting closer to the patient, thereby creating opportunities for a closer relationship. Being able to share a part of what is considered private also meant finding similarities between oneself and the patient. Elucidating a common responsibility to solve the situation also created the right conditions for getting to know each other well. One carer said:

Sometimes we end up in conflict, him and me. But in the end, we are all not so different and we are in this together. There are times when you do not have to be so professional and can let go a little of yourself as well in order to get to know one another.

### Being trustworthy

Caring for individuals in a forensic setting means being trustworthy and caring together—being dependent on each other. Facing patients who threaten harm and expose shortcomings forces carers to take a temporary step back to endure. Returning with new strength meant having an understanding, unravelling expressions of suffering and being able to both express trust and invite the patient to share and handle emotions. Developing trust is intertwined with being together with others, meaning being accepted as human despite one’s shortcomings. Being able to handle a stressful situation and set an example means being a role model for the patient.

There is a patient who has an ability to press my buttons, who sort of crawls under my skin. It is often about threats about me as a person, very unpleasant … Then I need to remind myself that we are all human, he is probably afraid. I´ve been in this situation before, and I feel relatively secure in such situations. I want him to be able to come and share his emotions with me even if they are expressed that way … hopefully he can learn from me.

### Maintaining stability in the community

This theme describes carers’ emotions in relation to the community when being and staying in a stressful situation. The ward is referred to as a community, which consists of the carers themselves, the patients and other carers all sharing fellowship with a common “we.” Carers’ lived experiences with the patients’ actions and expressions influenced the community and the individual carer. Maintaining peace and harmony in the community highlighted the importance of a caring climate for carers. Being part of the community meant feeling safe, persevering and being accepted for who you are. The community is described as a place where carers and patients come together as one group, which is further described in the two subthemes “Being perceptive to the dynamics in the community” and “Being a safe companion.”

### Being perceptive to the dynamics in the community

The subtheme being perceptive to the dynamics in the community included being observant and having a sense of control. Carers illuminated this by either staying in the moment or taking a step back and leaving the situation. Remaining present and keeping calm in a threatening situation is characterized by the interplay between the carer, patients and other carers. Being present meant becoming aware of the patients’ actions as well as the reactions of other carers, which elucidated the possibility of stress that could endanger the balance in the ward.

Sometimes, the only right thing to do is to take a step back and leave the situation for a moment. Otherwise, the whole ward becomes chaotic … there is a sense of security when it becomes predictable.

Being perceptive means becoming aware of the responsibility one has to others in the community, this is intertwined with being in control and having a sense of safety and security. Such an awareness occasionally means controlling chaotic feelings, trying to overcome unwanted emotions and not being part of an escalating situation.

I want to prevent the situation from escalating, from escalating to violence. If I get stressed, then I lose control. I think my need to get control of the situation makes me focused and confident, and I cannot handle it if I let that stress or fear take over.

### Being a safe companion

Regulating oneself means being a safe companion, stepping forward and taking responsibility for the stress experienced by other members of staff in a threatening situation. As carers described, handling responsibilities is intertwined with being someone to turn to, being able to carry the vulnerabilities of others, being attentive, and interpreting and alleviating the emotions of other carers when finding them in distress. Having the courage to relieve others stems from a sense of feeling confident in oneself.

I feel that I need to come to grips with this and her feelings so she can take a step back. It is important that you do not escape responsibility, that I am poised in myself to take that step forward. You want to help others yourself, and you do that by taking responsibility and relieving others.

Being reliable and accountable also means becoming aware of having the necessary tools to deal with the situation, remaining calm in the encounter and not acting out of affection. Elucidating the meaning to endure instead of abandoning the patient means staying present in the moment.

I cannot just crawl away or disappear or shout back. I have a responsibility in this. It is my responsibility to make sure that I have a strategy for dealing with this.

## Discussion and comprehensive understanding

Our results indicate that carers regulated themselves because they were emotionally touched by having to face their vulnerability as well as that of the patient, which proved to be a challenge when trying to maintain stability in the ward. Counteracting disorder and establishing order through finding balance and creating calm were seen as necessities to take care of oneself and protect the patient as well as the community. Regulating oneself helped carers create the conditions needed to allow them to achieve their goal; namely, to create alliances and a close relationship with patients, which made it possible to support the patient and achieve improved health. Despite being focused on oneself as a carer in the interaction with patients and other carers, all themes describe the fact that regulating oneself was used to achieve stability and create a predictable atmosphere and a caring environment.

***Preserving oneself as a carer*** refers to carers’ lived experiences of dealing with and overcoming what was described as unwanted and conflicting emotions. Being a carer means being emotionally flexible and sensitive when encountering patients and finding a sense of tolerance to solve conflicting situations which is also emphasized in other studies (Gildberg et al., 2021). Caring within a forensic setting also means feeling unsupported and unprepared at times while dealing with ongoing internal obstacles as well as feelings of fear and experiences of powerlessness when dealing with the task at hand, findings in line with Harris et al. (2015). This is because encountering a patient is often linked to facing danger and risk (Olsson et al., 2015), as caring in a forensic setting means having to make decisions regarding correction and discipline (Askola et al., 2016) while also experiencing stress and a fear of facing an inverted position of power that results in being victimized and threatened by the patients (Hellzen et al., 1999). Being in such a stressful and asymmetric environment where the main mission is to understand maladaptive behaviour, regulating emotions could be seen as a way to keep moving forward and gaining that crucial understanding (Benbouriche et al., 2016). Our findings suggest that finding the source of suffering together with the patient could prove to be a challenge, as being present in suffering means encountering one’s shortcomings (Vincze et al., 2015) because understanding oneself is intertwined with understanding the life of another (Rydenlund et al., 2019). Being a carer and having a person-centred attitude requires reflecting upon whether one is prepared to listen to the patient’s story, challenging one´s preconceived notions and being accessible to the patient (Rytterström et al., 2020).

Emotional regulation means not only turning the gaze inwards but also becoming aware of and managing conflicting emotions while not displaying the ongoing internal struggle in front of others, which coincide with Gross (1998). This involves challenging one’s understanding of oneself and preunderstanding to have an openness to the patient’s lifeworld, thus maintaining a caring attitude (Hörberg, 2018). In contrast, not being able to reassess conflicting emotions and suppressing contradictory feelings instead results in distancing oneself from the patient and self-criticism due to a lack of self-compassion, criticizing oneself and using regulating oneself for avoidance (Oostvogels et al., 2018).

Our findings also suggest that carers used the strategy of regulating themselves to ***build an alliance with the patient***. Meaning adapting one’s emotions to the patients’ expressions of suffering was made possible by opening up, showing a part of what was considered personal, letting down the façade and revealing a part of one’s vulnerability. The interaction between carer and patient is central to understanding and approaching patients’ suffering in forensic psychiatry care (Salzmann-Erikson et al., 2016). The carer’s emotional presence and interest in being involved in the patient’s lifeworld were emphasized by Vincze et al. (2015). If the carer is prepared to see the patient as a person and encounter the patient’s lifeworld, opportunities are created for viewing what is considered personal and gaining a deeper understanding of the patient’s vulnerability and lifeworld (Hörberg, 2018). Being present with someone means being accessible and available with one’s whole person—a presence that means an adaptation to the unique situation (MacKinnon et al., 2005). Being present enables the carers to accomplish their caring obligation of alleviating suffering (Rydenlund et al., 2019). The findings advocated that carers must possess personal and professional maturity and base their practice on the moral principles of commitment and respect for the patient as a unique individual. If the carer’s presence is accepted by the patient, a carer may be able to share the patient’s suffering, thereby creating a place where the patient could be in contact with their suffering, share it with another human being and find a way to move forward (Fredriksson, 1999). An encounter can unite the carer and patient by sharing experiences and creating a sense of belonging (Rytterström et al., 2020). Our findings suggest that sharing means developing trust and is the very foundation upon which a fruitful relationship within a forensic setting is established. Carers talking about their emotions encourages the patients’ sense of participation (Söderberg et al., 2019). Forming a relationship is considered indispensable and a prerequisite for forensic psychiatric care (Kumpula et al., 2019). According to Gildberg et al. (2010) the carer-patient alliance is seen as a trust-building process towards health and recovery that takes place between carer and patient. Although conditions in forensic psychiatric care are and can be seen as paternalistic because they are affected by current regulations, the quality of daily care depends on the carer’s relational and personal characteristics (Gildberg et al., 2010). Maguire et al. (2014) emphasized the importance of the carer-patient alliance and a trusting relationship and highlighted the positive therapeutic results that such a person exhibits in a forensic psychiatric context compared to authoritarian methods as it may reduce aggressions, elicit cooperation and reduce coercive interventions.

The third and final theme, ***maintaining stability in the community***, reflects that self-regulation includes not only the relationship with the patient but also with other carers and the community they share. Maintaining a calm environment comes not only through interaction with oneself but also with patients and other carers, a finding previously proposed by Enarsson et al. (2017). Our findings suggest that carers regulate emotions to maintain a calm and stable environment, which was seen as favourable when carrying out caring actions and building a relationship with the patients. Stability is seen as a temporary condition in the community and is dependent on whether carers choose to be active or passive (Salzmann-Erikson et al., 2011). Taking responsibility, taking charge and being accountable for the relationship is of importance for carers (Kumpula et al., 2019) as well as having a sense of being in control (Hellzen et al., 1999) and feeling safe when performing caring actions (Gildberg et al., 2021). According to Salzmann-Erikson et al. (2011), preventing turbulence and maintaining and restoring stability is accomplished from nursing care, if the patient is not experiencing a state of well-being, stability will less likely appear. Stability is a complex state, as it is reliant on the actions of both patients and carers (Salzmann-Erikson et al., 2011). Our findings suggest that the community was something all parties shared, something they had in common. The building of a relationship starts with a conversation that stems from having something in common. It is the carer’s responsibility to invite the patient into a community where suffering can be experienced as enduring. When the conversation ends, the community remains in the sense of equality in being a human among other humans (Fredriksson, 1999).

Giving meaning to the patients’ expressions in the encounter means making the patients’ experiences part of the carers’ life experience (Rytterström et al., 2020). By accepting the patients’ worlds as part of their own, carers are also faced with a responsibility to “be there for the other” (Rydenlund et al., 2019), a notion that is intertwined with the findings of this study that emphasizes the importance of being a safe companion to maintain stability in the community. Finding a mutual sense of tolerance among colleagues prevents the individual carer too the use of restrictive and coercive actions (Gildberg et al., 2021). In cases when the patient’s expression is noticed as a fact and the carer does not engage in the patient’s expression, or if the carer does not care about what the patient is trying to convey and only states what the patient does or says as a fact, the patient’s actions or opinions are transformed into facts and the patient is objectified by means of these (Skjervheim, 1996). Our findings suggest that carers regulate themselves to maintain stability in the community, which is in line with Björkdahl et al. (2010) who states that a mutual perspective that emphasizes a person-to-person relationship together with the carer’s ability to humbly take a step back to prevent carers being drawn into situations in an uncontrolled manner (Björkdahl et al., 2010), the carer has an opportunity to avoid objectifying the patient. One way to deal with this is through regulating oneself and with the preservation of symmetry between the parties (Skjervheim, 1996). In this way, the patient is not seen as an object and the carer is less likely to only trusts rules, routines and norms in relation to the patient.

In summary, regulating oneself in meeting with patients in forensic psychiatric inpatient care means that carers face situations that threaten their professional identity and the stability of the department. By participating in the patients’ lifeworlds and considering the patients’ expressions, carers may be able to regulate their feelings and enable the alliance between themselves and the patients. For carers, the challenge is how professionalism (i.e., the norm of the good carer) plays into their interpretation of the situation. It is of clinical importance to allow carers to not only discuss and be educated in matters concerning the patients but also be guided regarding managing conflicting emotions and vulnerabilities and finding an approach that allows a permissive climate of self-reflection in everyday encounters. The challenge is for the carer to implement a life-world-based approach to care and learning that can lead to strategies that support them to open up to the patient’s lifeworld, a strategy that has the potential to strengthen the patient’s health and learning processes. Such an approach requires sensitivity and critical assessment, and as Lögstrup (1997) emphasized, it is not enough to know the requirements, norms and rules because they rarely give satisfactory and unambiguous answers. Our interpretation was that regulating oneself was used to cope, de-escalate a stressful situation, find strength and create a caring environment to enhance recovery for the patients.

### Limitations of the study

All the interviews were conducted digitally due to the ongoing pandemic, which may have prevented the participants from speaking freely and feeling comfortable depending on their habits of communicating through digital media. However, all the interviews were conducted with the use of visual aids, which enabled the first author to interpret facial expressions and made it easier to understand the participants’ emotions. To ensure trustworthiness, participants must speak as truthfully as possible regarding their lived experience (Lindseth & Norberg, 2004); therefore, it could be viewed as a strength that the first author was known to most of the participants as a former colleague or tutor, having prior experience of the studied context. This hopefully enabled the participants to speak honestly and with trust and not be hesitant and afraid of revealing their shortcomings. However, this could also mean that the participants refrained from revealing weaknesses. Being known to the participants could, in one sense, mean gaining access to sensitive information and could also imply that the interviewer, the first author, became insensitive to conditions and circumstances that seemed obvious. Thus, this may have created a challenge when problematizing one’s preunderstanding and the interviewer could have missed hidden messages during the interviews. This was an ongoing process that forced the first author to challenge their preunderstandings to be able to discover underlying and unspoken meanings. The first author conducted and transcribed all the interviews, but all authors contributed to this study. The transcribed interviews were read by all authors, which resulted in enriching discussions that further helped the first author to curb their preunderstandings. Not all the authors had prior experience in the studied context, which was seen as a strength as it allowed the text to be viewed from a different standpoint. Notably, the participants’ professions and education levels differed, something that may have influenced their interpretation and understanding of regulating oneself. However, all participants in this study practised nursing close to patients and their experiences of regulating themselves are relevant and provide the knowledge that this study sought.

Regulating oneself arose because of encounters and circumstances with patients who were perceived as threatening and/or violent or annoying or encounters during which carers perceived themselves as annoying. It should be noted that the participants’ narratives tended to depict forensic psychiatric care as dominated by demanding situations. This could be because interviews started with a short presentation of a prior study (Hammarström et al., 2019) that could have influenced the participants’ focus on the negative part of encounters. Conducting this study means as Lindseth and Norberg (2021) states, the interpreter interprets the text and the text interprets the interpreter. To establish rigour in the structural analysis, emphasis has been on the shift to a more phenomenological attitude as the text was viewed and discussed as objectively as possible, in line with Lindseth and Norberg (2004) who states that during this part of analysis it is of importance to consider the text as independently as possible from its context. The findings of this study do not present an absolute truth, instead the findings should be perceived as an interpretation of the lived experience of regulating oneself when caring for patients with mental illnesses in forensic inpatient care because a text can be interpreted in numerous ways (Ricoeur, 1976). Our analysis was looking for depth, nuances and meanings of experiences in individuals, and the findings can thus not be generalized to a population or be representative of a context (Patton, 2014). At the same time, we intended to contribute with a perspective from a limited range of participants that can be brought into dialogue with already existing perspectives in the field. Humans and their phenomena are contextual and whether knowledge can be said to have transfer value will depend on the situation or relationship to which the knowledge will be related. Attempts have been made to secure the transfer value of this study with rich and transparent descriptions of the method, participants and context so that readers are able to decide whether the findings are transferable to another setting. The transferability of the findings may be affected by the fact that laws and regulations governing forensic psychiatric care vary internationally. However, the findings are not considered exclusive to the studied context, as encounters with patients, handling and regulation of emotions are relevant to most carers in other contexts as well.

## Conclusion

Regulating oneself meant acknowledging and understanding one’s shortcomings and vulnerability, which proved to be a prerequisite for getting to know the patient. The strategy of regulating emotions and reactions was used to create the conditions for a caring climate and preserve the stability of the ward. Relating to and dealing with emotions appeared to be a necessary element in forensic care, which can have negative consequences for both patients and carers, if not addressed. There is therefore a need for increased knowledge about the regulation of emotions and how it can assist carers in their daily work. Also, about what support and facilitation carers need to develop such skills.

### Relevance to clinical practice

Our findings emphasize the importance of carers having a strategy to act upon when establishing relationships and alliances in encounters with patients in forensic psychiatry. Addressing the carers emotions and being allowed to verbally discuss challenging encounters and their shortcomings seemed to be a necessity. Thus, to face and fully comprehend expressions of suffering, carers must become aware of their vulnerabilities so they do not project them onto others, which could impair establishing relationships with patients and fulfiling their caring tasks. Instead, carers must engage in self-reflection and find the courage to grasp suffering and allow themselves to be emotionally touched to regulate themselves when needed. Carers may need support and facilitation to understand and process their own feelings in professional work. We suggest that efforts should be directed towards such facilitation, which is aimed at both education and organizational level in the service.
